# Zooming into the Complex Dynamics of Electrodermal Activity Recorded during Emotional Stimuli: A Multiscale Approach

**DOI:** 10.3390/bioengineering11060520

**Published:** 2024-05-21

**Authors:** Laura Lavezzo, Andrea Gargano, Enzo Pasquale Scilingo, Mimma Nardelli

**Affiliations:** 1 Dipartimento di Ingegneria dell’Informazione, University of Pisa, 56122 Pisa, Italy; andrea.gargano@phd.unipi.it (A.G.); enzo.scilingo@unipi.it (E.P.S.); 2 Research Center “E. Piaggio”, University of Pisa, 56122 Pisa, Italy

**Keywords:** multiscale, fractality, electrodermal activity, ComEDA, emotions, affective computing, CASE dataset, AMIGOS dataset

## Abstract

Physiological phenomena exhibit complex behaviours arising at multiple time scales. To investigate them, techniques derived from chaos theory were applied to physiological signals, providing promising results in distinguishing between healthy and pathological states. Fractal-like properties of electrodermal activity (EDA), a well-validated tool for monitoring the autonomic nervous system state, have been reported in previous literature. This study proposes the multiscale complexity index of electrodermal activity (MComEDA) to discern different autonomic responses based on EDA signals. This method builds upon our previously proposed algorithm, ComEDA, and it is empowered with a coarse-graining procedure to provide a view at multiple time scales of the EDA response. We tested MComEDA’s performance on the EDA signals of two publicly available datasets, i.e., the Continuously Annotated Signals of Emotion (CASE) dataset and the Affect, Personality and Mood Research on Individuals and Groups (AMIGOS) dataset, both containing physiological data recorded from healthy participants during the view of ultra-short emotional video clips. Our results highlighted that the values of MComEDA were significantly different (*p*-value < 0.05 after Wilcoxon signed rank test with Bonferroni’s correction) when comparing high- and low-arousal stimuli. Furthermore, MComEDA outperformed the single-scale approach in discriminating among different valence levels of high-arousal stimuli, e.g., showing significantly different values for scary and amusing stimuli (*p*-value = 0.024). These findings suggest that a multiscale approach to the nonlinear analysis of EDA signals can improve the information gathered on task-specific autonomic response, even when ultra-short time series are considered.

## 1. Introduction

Over the last few decades, the scientific literature has provided enormous support for the thesis that complexity arises in the behaviour of biological systems, with processes developing in an intricate network of interactions and feedback loops on different space–time scales [1,2,3,4]. This motivated the necessity of assessing these phenomena on several temporal scales [5], in order to distinguish systems that are really “complex”, i.e., systems exhibiting similar behaviour on multiple scales, from noisy systems that are chaotic and highly unpredictable only on the first time scale [6]. The mathematical concepts of fractality and time-scaling have been used as a hallmark of physiological dynamical system analysis, and the multiscale view allowed increasing the capability of chaos-theory-derived metrics in characterizing specific healthy and pathological states of the central and autonomic nervous systems [7,8,9,10]. Indeed, previous studies already reported on successful applications of multiscale metrics extracted from Heart Rate Variability (HRV) [2,11,12,13], electroencephalographic [14,15], and electromyographic [16] time series.

Among the plethora of physiological signals recorded non-invasively by wearable sensors, the electrodermal response provides relevant information about autonomic modulation. In particular, electrodermal activity (EDA) is the measurement of alterations of the electrical properties of the skin, a process controlled by the sympathetic nervous system [17,18]. An increase in sympathetic activity causes a subsequent increase in the EDA signal amplitude [19], leading to high confidence in its use as a biomarker for physiological arousal [18]. For this reason, EDA features computed through time and frequency analysis of EDA signals have been widely used to distinguish between different levels of arousal in emotional responses [20,21,22,23].

Recognizing emotions is one of the aims of the burgeoning research field of affective computing [24]. The assessment of the personal emotional state can be used in several real-world applications [25], from clinical evaluation of emotional dysregulation [26] to the development of software interfaces that adapt to the user’s emotional state, improving its engagement in education and work activities [27]. The monitoring and processing of physiological signals is a promising tool for the objective assessment of emotional changes. Self-evaluation of the emotional state could be biased by several aspects, e.g., personality traits, and presents several limitations in its applicability in experimental and naturalistic contexts [28]. Therefore, previous literature in the affective computing field proposed several approaches to develop computational models for emotion recognition using physiological time series [20,21,22,29]. The majority of these studies were based on assessing changes in the autonomic nervous system (ANS) activity, which can be observed through inexpensive and non-invasive sensors [30]. Specifically, as demonstrated by the experimental results obtained from HRV analysis [31,32,33], the investigation of nonlinear autonomic dynamics is fundamental for the assessment of emotions and moods.

However, while numerous techniques derived from chaos theory have been successfully applied to the characterization of HRV dynamics, only a few studies have investigated the complexity of the EDA [34]. The reason for this lack of application is especially linked to methodological issues, since the reconstruction of the phase space, the approach on which several nonlinear time series analysis techniques are based, presents important limitations if the mean and standard deviation of the starting time series fluctuate significantly, as in EDA signals. In [35], we proposed *ComEDA*, an algorithm for EDA complexity level estimation, based on a novel way to characterize the dynamics in the phase space, which overcomes the issues related to EDA signal fluctuations. This metric was reported to be reliable in ultra-short (i.e., less than or equal to two-minute-long recordings) time series analysis and efficient in recognizing physical and mental stress [35]. Unlike the standard EDA features, which are strongly linked to the arousal dimension, the results obtained with *ComEDA* confirmed the hypothesis of a greater task-specificity of nonlinear metrics, as already advanced in [34]. Our approach was specifically designed to account for the characteristics of the EDA, thus addressing the limitations of standard entropy-based methods for physiological time series, e.g., Approximate Entropy [36] and Sample Entropy [37]. In particular, the use of angular distances in the phase-space computations instead of the Chebychev distances allowed the challenge posed by the spiky nature of the EDA signal amplitude to be addressed. Furthermore, as we took advantage of the entire information present in the pair of points in the phase space via the computation of its probability density function, our approach can be considered threshold-free. Finally, ComEDA has proven to be reliable even within ultra-short windows [35], rendering the metric well-suited for multiscale analyses that entail a reduction in the number of points as the time scale increases. In this study, we propose a further step towards the complete characterization of the complex properties of EDA and present a multiscale version of the *ComEDA* algorithm, i.e., Multiscale ComEDA (*MComEDA*). We expand upon our previous results in [35] by analysing the behaviour of the EDA signal at a multiscale level, aiming to investigate the EDA characteristics in response to emotion-specific stimulation, considering its fractal properties. We tested the novel metric *MComEDA* on real EDA recordings of two publicly available datasets, i.e., the Continuously Annotated Signals of Emotion (CASE) dataset [38] and the Affect, Personality and Mood Research on Individuals and Groups (AMIGOS) dataset [39], both containing physiological signals of healthy subjects recorded during video-based emotional stimulation, the most used emotion induction paradigm in affective computing studies [40]. Statistical tests highlighted differences between *MComEDA* and *ComEDA* values among four different types of emotional stimuli in the two datasets under investigation. The proposed measure and the experimental protocols are described in Section 2. The results are depicted in Section 3, with Discussion and Conclusions following in Section 4.

## 2. Materials and Methods

**Figure 1:**
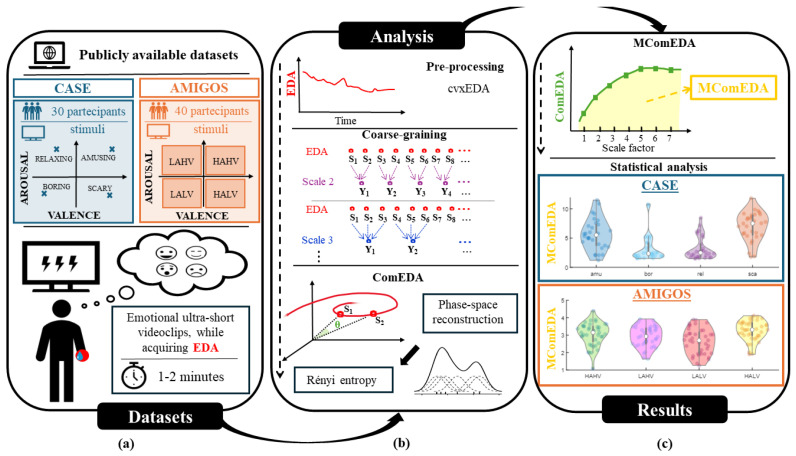
Flow–chart representing the comprehensive framework of the study design. Section (**a**) illustrates the two publicly available datasets employed in the analysis (top), along with a representation of the stimulation type (bottom). In section (**b**), the principal steps of the algorithm utilized for the computation of the MComEDA index are provided. Lastly, section (**c**) portrays the MComEDA index derived from the multiscale trend, computed as the area under the curve (top). A brief overview of the outcomes of the statistical analysis is also presented.

The performances of MComEDA and ComEDA algorithms were tested on two different publicly available datasets, i.e., CASE and AMIGOS [38,39], containing EDA signals acquired during short and ultra-short video-based stimuli presented to healthy participants. A summary of the steps is graphically represented in Figure 1.

### 2.1. The Continuously Annotated Signals of Emotion (CASE) Dataset

Thirty healthy volunteers participated in this study, with a balanced gender design (50% females, females aged 25.7 ± 3.1 years, males aged 28.6 ± 4.8 years) [38,41]. The experimental protocol followed a within-subjects design with each participant viewing eight short emotion-inducing video clips whose duration ranges in [119; 197] s. The eight emotion-inducing video clips were selected according to the outcomes of a pre-study session [38], to induce four different emotions: fear, amusement, relaxation, and boredom. Two different video clips were used to elicit each emotion. The experimental protocol involved the administration of low-arousal stimulations, with the aim of inducing states of low activation, such as boredom and relaxation. This was achieved through the presentation of short clips of documentary excerpts and tropical scenes accompanied by relaxing music. Conversely, high-arousal stimulations, such as fear and enjoyment, were presented to induce feelings of high activations via short clips of horror and comedy movies, respectively. The presentation order of the videos was pseudo-randomized, thus creating a unique video sequence for each participant and avoiding any reproducible carryover effect due to the presentation of the same sequence of stimuli. In addition, a two-minute long blue screen video was shown to separate two consecutive video stimuli as a resting phase, allowing participants to recover in between each stimulation session. The self-assessment of emotions was collected in real time while each emotion-eliciting video was displayed to the participant. In particular, each participant was provided with a custom joystick interface designed for emotion assessment in a bidimensional space of valence and arousal dimensions, according to Russell’s bidimensional model of affects [42]. Statistical results reported in [41] confirmed that different emotional stimulations have indeed occurred based on the analysis of the continuously annotated data of emotion ratings.

A set of physiological signals was recorded and collected during the experimental session, as detailed in [38]. These included the electrocardiogram, recorded using three electrodes placed on the chest; the respiratory signal, acquired via a respiration belt placed on the high part of the torso; the photoplethysmographic signal, using a blood volume pulse sensor placed in the middle of the non-dominant hand; the skin temperature, measured with an epoxy rod thermistor on the pinky finger of the non-dominant hand; and the surface electromyographic signals of three facial muscles (i.e., zygomaticus major, corrugator supercilii, and trapezius) measured with bipolar configuration. The physiological data were acquired through a Data Acquisition system, whose sampling frequency was set to 1000 Hz. EDA signals, in particular, were acquired from index and ring fingers of the participants’ non-dominant hand, with Ag/AgCl electrodes fixed at the fingertips in bipolar configuration with finger straps using a Thought Technology galvanic skin response sensor. In this work, we rely on the EDA data of all eight emotion-eliciting videos to investigate the capability of this physiological signal to distinguish between 4 video-based emotional stimuli with different arousal and valence content.

### 2.2. The Affect, Personality and Mood Research on Individuals and Groups (AMIGOS) Dataset

The AMIGOS dataset [39] contains data from forty healthy volunteers watching emotional videos (13 females, age range between 21 and 40 years), in both solitude and group contexts. In this analysis, only the subset concerning the stimulation presented in the solo-viewing condition has been considered, to allow a fair comparison with the CASE dataset.

In the AMIGOS experimental protocol, each participant viewed 16 short videos which presented a duration in the range [51–150] s. These video clips were taken from specific databases already validated in previous studies, e.g., the DECAF [43] and MAHNOB-HCI [44] databases, and were previously annotated using the arousal and valence dimensions. Four videos for each quadrant of the valence–arousal space were presented in random order. The four quadrants were labelled according to the corresponding high or low arousal and valence levels, as follows: HVHA, HVLA, LVHA, and LVLA (where H, L, A, and V stand for high, low, arousal, and valence, respectively). Three videos for each quadrant of the arousal–valence plane were taken from the set of videos of DECAF database, which was based on the scoring of arousal–valence dimension of 72 volunteers; one additional video for each quadrant was selected from the MAHNOB-HCI database. All stimuli were excerpts from renowned movies, selected to elicit emotions in the four quadrants. For instance, to elicit LVHA, i.e., a highly arousing but unpleasant state, segments of famous horror movies were used, while, to elicit HVHA, i.e., a highly arousing and pleasant state, comedies were employed. The total timeline of the AMIGOS experimental protocol consisted of 16 trials, each one composed of a 5 s long baseline recording in which a fixation cross was shown, the presentation of one video clip, and the collection of the answers to questionnaires on participant’s self-assessment of valence, arousal, dominance, liking, and familiarity on a scale from 1 to 9 using a moving slider. In this study, we used the EDA signals provided by the AMIGOS dataset, recorded using the Shimmer 2R platform (at a sampling rate of 128 Hz), with two electrodes placed on the middle phalanges of the middle and index fingers of the left hand. We aimed to use the *ComEDA* and *MComEDA* metrics to discern the ANS response to video stimuli belonging to the four quadrants of the valence–arousal plane.

### 2.3. EDA Signal Pre-Processing

For both CASE and AMIGOS datasets, we used the same procedure for the pre-processing of EDA signals, based on the application of cvxEDA algorithm [22]. The EDA signal consists of the superposition of two different physiological components: the tonic and the phasic components. Whereas the former component is the baseline level containing only slowly varying spontaneous fluctuations related to the human overall psycho-physiological state, the latter represents the rapidly occurring variations of the skin conductance due to stimulus-specific reactions as well as non-specific ones. The cvxEDA approach models the skin conductance as the sum of the two main components, i.e., the tonic and phasic, and an additive noise term. In more detail, the cvxEDA algorithm recasts the problem of estimating the tonic and phasic components of the EDA into a convex optimization problem, accounting for the presence of artefacts, modelling, and measurement errors, and taking advantage of sparsity as well as a physiologically inspired description of the sweat diffusion mechanism inducing skin conductance variations [22]. After the application of cvxEDA, the cleaned EDA signal is obtained by the sum of the tonic and phasic components.

A frequency rate of 5 Hz was used for all the EDA signals used in this study. Then, the downsampled signals were normalized using the z-score method.

### 2.4. The Multiscale ComEDA Approach (MComEDA)

The *ComEDA* index [35] was developed to quantify the complexity of the EDA nonlinear dynamics. The first step of this algorithm is the phase-space reconstruction, which consists of the reconstruction of the attractor related to the EDA time series under investigation. Specifically, according to Takens’ theorem [45], it is possible to embed each time series in a multidimensional space by computing the so-called embedding parameters: the time delay (τ), which is the lag value to plot each time series against itself, and the embedding dimension (*m*), which determines the phase-space dimensionality. We calculated the values of the optimal embedding parameters according to the state-of-the-art approaches. In more detail, the value of τ is computed as the first local minimum of the auto mutual information function, a nonlinear generalization of the autocorrelation function [46,47]. Moreover, the *m* value was selected according to the False Nearest Neighbors (FNN) approach [47,48], which studies changes in distance between neighbouring points in the reconstructed phase space, as the original time series is progressively embedded by increasing the dimension. For each EDA time series we calculated the two parameters, i.e., τ and *m*, using the implementation presented in [47]. Given a generic *N*-dimensional time series [y(1),y(2),…,y(N)], the procedure for the attractor reconstruction results in the computation of the n=N−(m−1)τ embedded vectors. In particular, the generic *j*-th embedded vector is defined as follows:

(1) Y(j)=[y(j),y(j−τ),y(j−2τ),…,y(j−(m−1)τ)]

Given the embedded vectors, the distance between them can be computed. The ComEDA approach defines this distance (*d*) as the angular distance between each pair of vectors (Y(i),Y(j)), for 1≤i,j≤n, excluding self-matches, computed as follows:

(2) $$d_{ij}=cos\left(\theta \left(Y\left(i\right),Y\left(j\right)\right)\right)=\frac{Y(i)\cdot Y(j)}{{∥Y(i)∥}_{2}{∥Y(j)∥}_{2}}$$

in which · is used for the inner product and $\|\;{\|}_{2}$ for the Euclidean norm.

From the distance value $d_{ij}$, the probability density function (PDF) is computed by using a kernel density estimator based on linear diffusion processes with a Gaussian kernel [49]. The optimal number of bins (B) to compute the PDF is chosen according to the Sturges method [50]. Then, the quadratic Rényi entropy formula is applied [51], using the probability of each bin ($p_{i}$):

(3) $$R_{2}=-log_{2}\left(\sum_{i=1}^{B}p_{i}^{2}\right)$$

Finally, the value is standardized to the range [0, 1] according to the following equation:

(4) $$ComEDA=-\frac{1}{log_{2}B}R_{2}$$

In this study, we extended the application of *ComEDA* algorithm to perform the multiscale analysis of EDA response. Specifically, we introduced a first step at the beginning of the pipeline to compute the scaled time series from each original EDA signal, according to the coarse-graining procedure described below [2]. Starting from the original time series y=y(1),y(2),…,y(N), consecutive coarse-grained series $x^{\left(\beta \right)}$ are constructed, considering a specific range of values for the scale factor β. Each element of the β-coarse-grained time series $\left[x^{\left(\beta \right)}\left(1\right),x^{\left(\beta \right)}\left(2\right),\ldots ,x^{\left(\beta \right)}\left(\frac{N}{\beta }\right)\right]$ is computed using the following formula:

(5) $$\begin{array}{c} x{\left(i\right)}^{\left(\beta \right)}=\frac{1}{\beta }\sum_{b=(i-1)\beta +1}^{i\beta }y\left(b\right)\\ where\;1\leq i\leq \left\lfloor \frac{N}{\beta }\right\rfloor \end{array}$$

These new coarse-grained time series are iteratively used as input for the *ComEDA* algorithm, maintaining the values of τ and *m* parameters as the ones computed for the original time series. In this way, a value of ComEDA is obtained for each scale factor β, defining a multiscale trend. The MComEDA value is then derived from this trend computing the area under the curve of ComEDA as a function of β values.

In this study, the noiseless EDA signals, obtained after the cvxEDA procedure, were used as input for the MComEDA algorithm. The multiscale trends were extracted considering the range β∈[1–20] for the signals of the CASE dataset. Concerning the AMIGOS dataset, due to the shorter average length of the signals, the scale range was reduced to β∈[1–10]. The choice of the maximum scale factor (β) has been based upon the available data points subsequent to the final coarse-graining iteration. Specifically, a value of β = 20 has been adopted for the CASE dataset to ensure a minimum of roughly 30 samples at the most granular time scale. Conversely, for the AMIGOS dataset, a value of β = 10 has been employed to guarantee a minimum of roughly 25 samples across the highest scale range.

We performed all the procedures of pre-processing, *ComEDA*, and *MComEDA* computation in MATLAB (R2021b, Mathworks Inc., Natick, MA, USA).

### 2.5. Statistical Analysis

For each subject of the CASE dataset, we computed two values of each EDA complexity metric (*ComEDA* and *MComEDA*) for a single emotion, considering that the experimental protocol included two video clips for the same emotion type. Then, the complexity values related to the same emotion were averaged, obtaining four different values for each metric and each subject, corresponding to the four different elicited emotions: scariness, amusement, relaxation, and boredom.

Concerning the AMIGOS dataset, each subject was monitored during the elicitation through four emotional videos for each quadrant of the valence–arousal plane: High Arousal and Low Valence (HALV), High Arousal and High Valence (HAHV), Low Arousal and Low Valence (LALV), and Low Arousal and High Valence (LAHV). We computed the corresponding values of *ComEDA* and *MComEDA* and we calculated their average considering each quadrant separately. In this way, we obtained four different values for each metric and each subject, corresponding to the four different quadrants of the valence–arousal plane.

The statistical differences in the values of ComEDA and MComEDA metrics computed from the EDA signals acquired during different stimulation types (the four emotions for the CASE dataset and the four valence–arousal quadrants for the AMIGOS dataset) were subsequently investigated. The Lilliefors test [52] was used to assess the Gaussianity of the distributions (α=0.05), resulting in non-Gaussian distributions for the majority of cases. Afterwards, considering each dataset separately, we performed the non-parametric Friedman’s test to investigate differences in EDA complexity values among the four types of emotional stimulations. When the outcome pointed at statistically significant differences (*p*-value of Friedman’s test lower than 0.05), we conducted the post hoc analysis through the application of Wilcoxon signed-rank test for paired data. The Bonferroni’s correction of *p*-values was applied when testing for multiple comparisons [53].

### 2.6. Comparison with Standard Analysis: The EDASymp Index

The performance of the proposed index in highlighting different emotion-related autonomic changes was compared with one state-of-the-art metric for EDA assessment: EDASymp [54], which is an index of sympathetic activation. EDASymp is defined as the mean value of EDA power spectral density within the frequency range [0.045–0.25] Hz. EDASymp was computed for each stimulation over 30 s windows, with no overlap, and then averaged to obtain a single index. Subsequently, the two EDASymp values obtained for the stimulation of the same kind were averaged and the statistical analysis was performed following the same procedure described above.

## 3. Results

The statistical results obtained from the application of the *ComEDA* and *MComEDA* approaches to the CASE and AMIGOS datasets are reported below.

### 3.1. CASE Dataset

**Figure 2:**
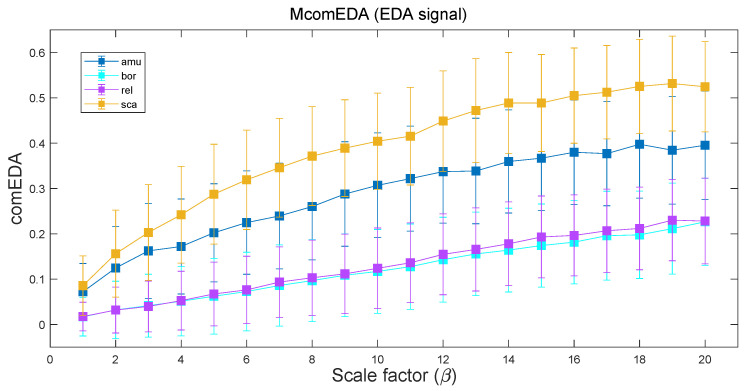
Trends of ComEDA as a function of β related to the EDA signals of the CASE dataset. For each scale factor β, the ComEDA values of the 30 participants were represented in terms of median ± MAD (Median Absolute Deviation) according to the type of emotional stimulus.

Figure 2 depicts the median trend of ComEDA values as a function of the scale factor β, in the range [1,20], for the four different emotional stimulations, i.e., boring, relaxing, scary, and amusing. As highlighted by the trends, when β increases, the separation between stimulations for high-arousal emotions (i.e., amusing and scary) and the low-arousal emotions (i.e., boring and relaxing) becomes clearer. Above β>8, even the statistical difference between the scary and amusing stimulations becomes discernible (*p*-value < 0.05), whereas no distinction is shown between the low-arousal stimuli.

**Figure 3:**
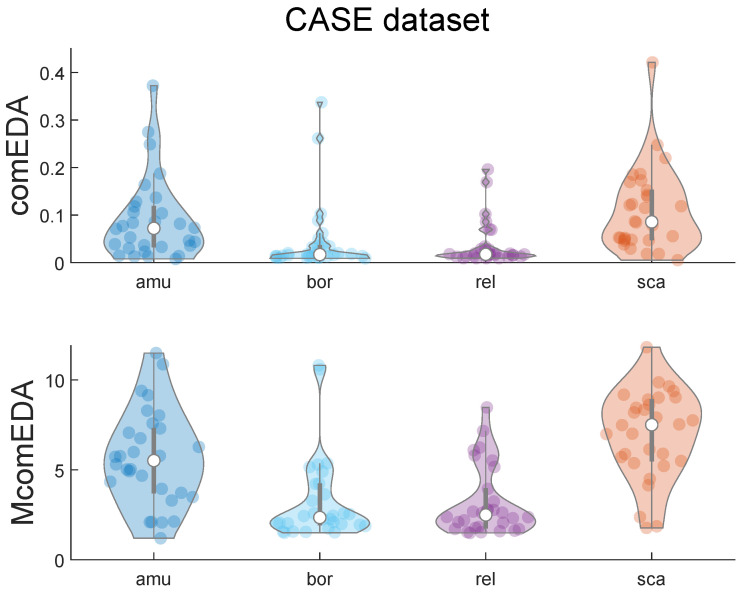
Violin plots highlighting the dispersion of the ComEDA (**top**) and MComEDA (**bottom**) values computed for the noiseless EDA signals per each emotion of the CASE dataset. The values reported correspond to the mean values of the complexity indexes, computed by averaging for the same participant the two complexity indexes calculated from the time series of the two videos (i.e., the two videos for each induced emotion). The emotion labels are shortened with the following convention: “amu” = amusement, “bor” = boredom, “rel” = relaxation, and “sca” = scariness.

MComEDA values were computed as the area under the curve of the trends reported in Figure 2. The violin plots shown in Figure 3 report on the distributions of the ComEDA and MComEDA values related to the four emotions of the CASE dataset. For both the complexity metrics, low-arousal stimuli (boring and relaxing) result in lower values of complexity if compared to high-arousal video clips (amusing and scary).

A *p*-value of 1.893 × 10^−10^ for *MComEDA* and 2.280 × 10^−8^ for *ComEDA* resulted from Friedman’s test. Table 1 summarizes the results of the Wilcoxon test applied to compare each pair of emotions for both ComEDA and MComEDA, after Bonferroni’s correction of *p*-values.

Concerning the statistical results related to the ComEDA values (see Table 1—right), the single-scale approach distinguished between high- and low-arousal states, finding statistically relevant differences (*p*-value < 0.01) in these comparisons. However, the comparisons between stimuli belonging to similar arousal categories, i.e., the two low-arousal stimuli (boring vs. relaxing) and the two high-arousal stimuli (scary vs. amusing), did not highlight statistically significant differences (*p*-value > 0.05).

In contrast, with the MComEDA metric (see Table 1—left) we were also able to distinguish between the two different high-arousal stimulation types, reporting a statistically significant difference (*p*-value = 0.024) between the EDA complexity values related to amusing and scary stimuli. This result is coherent with the trends shown in Figure 2, where this difference unfolds only as β increases. However, the difference between the low-arousal stimuli (boring vs. relaxing) was not statistically significant (*p*-value > 0.05).

### 3.2. AMIGOS Dataset

**Figure 4:**
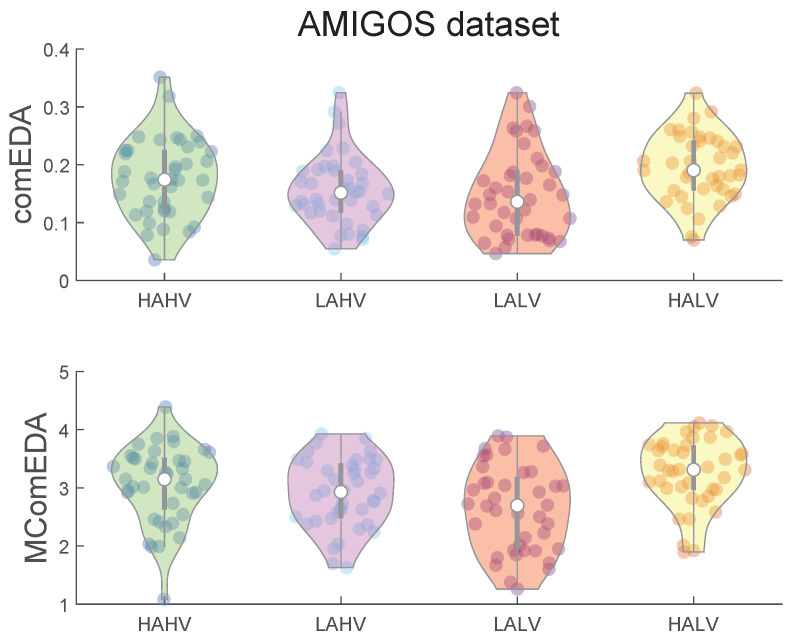
Violin plots highlighting the dispersion of the ComEDA (**top**) and MComEDA (**bottom**) values computed for the noiseless EDA signals per each emotion of the AMIGOS dataset. The values reported correspond to the mean values of the complexity indexes, computed by averaging for the same participant the four complexity indexes calculated from the time series related to the same valence–arousal quadrant. The quadrants are shortened with the following convention: “H” = High, “L” = Low, “A” = Arousal, “V” = Valence (e.g., HALV refers to the quadrant of high arousal and low valence).

When we analysed the EDA signals of the AMIGOS dataset, we investigated the spatial complexity of the EDA dynamics in healthy subjects responding to emotional stimulation belonging to the four quadrants of the arousal–valence plane, i.e., High Arousal and Low Valence (HALV), High Arousal and High Valence (HAHV), and Low Arousal and Low Valence (LALV), Low Arousal and High Valence (LAHV). Figure 4 shows the violin plots related to the ComEDA and MComEDA values for the four types of emotional stimuli. For both metrics, consistently with the results on the CASE dataset, complexity values were lower for low-arousal videos than for high-arousal stimuli.

After applying Friedman’s statistical test, we obtained the following *p*-values: 5.144 × 10^−4^ for *ComEDA* and 0.001 for *MComEDA*. Table 2 summarizes the *p*-values resulting from the multiple comparisons using the Wilcoxon test after Bonferroni’s correction. The right table presents the ComEDA performance: a statistically significant difference (*p*-value < 0.01) was found between the HALV state and the two low-arousal states, LAHV and LALV. The other comparisons did not show statistically significant differences. As reported in the left side of Table 2, the results of the MComEDA approach confirmed the results obtained with the single-scale metric, showing significantly higher values (*p*-value < 0.05) for HALV videos when compared to the low-arousal stimuli, additionally distinguishing between stimuli belonging to the HAHV and LALV quadrants (*p*-value = 0.007).

### 3.3. Comparison with EDASymp and Summary of Results

The results obtained applying the statistical tests to EDASymp values [54] are presented in Table 3, which contains the *p*-values obtained after Bonferroni’s correction. Concerning the CASE dataset, scary stimuli had significantly higher values with respect to relaxing stimuli (*p*-value < 0.01) while other comparisons were statistically non-significant. For the AMIGOS dataset instead, LALV had significantly (*p* < 0.05) lower values than LAHV and HALV.

We can summarize all the results obtained from the statistical analysis of standard and nonlinear EDA metrics for the two datasets as follows:

1. CASE dataset: The standard analysis with EDASymp allowed discriminating the EDA signals acquired during scary stimuli from the EDA signals related to boring videos. When we analysed the nonlinear dynamics of EDA, the complexity analysis using ComEDA at a single scale showed that amusing and scary stimuli were significantly different from boring and relaxing ones, whereas no significant differences were found comparing boring vs. relaxing or scary vs. amusing. Analysing the complexity of EDA signals at a multiscale level, we found other significant results in addition to the single-scale findings. In fact, the comparison between amusing vs. relaxing stimuli was also significant.
2. AMIGOS dataset:EDASymp values were statistically different when comparing LALV stimuli with LAHV and HALV. Considering the single-scale complexity analysis, HALV stimuli were significantly different from LAHV and LALV videos. Using MComEDA, the comparison between LALV and HAHV elicitation was also significant.

## 4. Discussion and Conclusions

This study presents *MComEDA*, a dedicated metric to assess the complexity of the EDA signals, accounting for multiscale physiological dynamics from a nonlinear time series analysis perspective. *MComEDA* expands on state-of-the-art techniques for the EDA spatial complexity quantification in the reconstructed phase space [35], by embedding a multiscale view of the system aiming at discerning subtle physiological changes. Specifically, we tested the applicability of this approach in emotion recognition case studies where a fundamental challenge lies in distinguishing nuanced emotional states.

We tested the capabilities of the *MComEDA* algorithm to discern the physiological responses to different categories of emotional video stimuli using two publicly available datasets, i.e., the CASE [41] and the AMIGOS [39] datasets. Then, we compared the performance of *MComEDA* with the results obtained applying the single-scale version of the algorithm, i.e., *ComEDA* [35]. In more detail, we investigated how the *ComEDA* and *MComEDA* metrics perform when setting as input the noiseless EDA signals (i.e., the sum of the tonic and phasic components after the application of the cvxEDA approach as a pre-processing step).

Concerning the first dataset (CASE), four specific emotions were elicited in a group of thirty healthy subjects, i.e., boredom, relaxation, fear, and amusement, elicited by short and ultra-short video clips. Notably, the results highlighted by the analysis of the CASE EDA signals via the MComEDA algorithm showed the capability of statistically distinguishing (*p*-value < 0.05) between all the pairs of different kinds of emotional stimuli, with the only exception given by the comparison between the two low-arousal stimuli (i.e., boring and relaxing), which was not significant.

In the case of the second dataset (AMIGOS), sixteen videos were presented to forty healthy subjects and categorized according to their membership of the four quadrants of the arousal–valence plane (HALV, HAHV, LALV, and LAHV). Using MComEDA, we found statistical differences between the stimuli in the HALV quadrant (*p*-value < 0.05) and the two low-arousal quadrants. In addition, the MComEDA values were statistically different when we observed the trends in the multiscale complexity of EDA signals recorded during HAHV and LALV stimuli (*p*-value < 0.05).

Compared to the single-scale approach of ComEDA, the here-proposed coarse-grained version of the algorithm showed a general performance improvement. In particular, considering the results of the CASE dataset, MComEDA distinguished between the two high-arousal stimuli (i.e., amusing vs. scary) whereas this nuanced difference was not detected for the single-scale scenario. As shown in the multiscale trend of Figure 2, the two high-arousal emotions corresponded to progressively different levels of complexity as the scale factor β increased, with this distinction being statistically significant (*p*-value < 0.05) for values of β above 8. Consistently, when analysing the results of the AMIGOS dataset, the multiscale approach allowed for a statistically significant distinction between the HAHV and LALV quadrants (*p*-value = 0.007), which was not possible if considering only a single time scale.

Regarding the comparison with standard metrics for EDA assessment, MComEDA outperformed EDASymp for both datasets. Concerning the CASE dataset, we found five out of six statistically significant comparisons with MComEDA, and only one by using EDASymp. For the AMIGOS dataset, both metrics were capable of distinguishing HALV from LALV. However, on the one hand, MComEDA allowed distinguishing HALV vs LALV and HAHV vs. LALV; on the other, EDASymp distinguished LALV vs. LAHV, suggesting the importance of considering both frequency and nonlinear metrics to characterize the phenomena from different perspectives.

In this work, the median MComEDA trends were comparable across both datasets under investigation, with high-arousal states being characterized by higher complexity levels. In particular, fear, an emotional state characterized by high arousal and low valence, was related to the highest values of MComEDA found for the CASE dataset, matching the behaviour of the HALV video stimuli for the AMIGOS dataset. Our findings on the higher levels of EDA complexity for strongly arousing emotional stimuli were in line with the hypotheses highlighted in previous literature [55], where a higher arousal level was associated with higher activation of the sympathetic nervous system which leads to more irregular and complex fluctuations in skin conductance. Even if the trends of the MComEDA values found a correspondence between the emotion categories of the CASE dataset and the valence–arousal quadrants of the AMIGOS dataset, the performance associated with the first experimental protocol is undoubtedly better than that of the second (five significant comparisons out of six for CASE, and three out of six for AMIGOS). One reason for this disparity in the discriminative power can be attributed to the different categories used to define the classes of emotional stimuli in the two datasets, which were also used during the application of statistical tests on complexity features. In more detail, in the CASE dataset, each of the four emotions was elicited with two different videos and the respective scores were proven to be annotated into a specific neighbourhood of the arousal–valence plane, ensuring that a precise target emotion was provoked (e.g., fear). However, for the AMIGOS dataset, the four videos for each category could be spanned across an entire quadrant (e.g., high arousal and low valence), therefore causing different emotions (e.g., fear, disgust, anger) to fall into the same broad category. In addition, the CASE dataset had longer recordings on average, allowing the calculation of 20 scales for the coarse-graining procedure whereas the AMIGOS dataset could not guarantee reliable results above 10 scales due to the shorter duration of its time series.

Building upon the previous affirmation, we highlight a limitation intrinsic to the multiscale approach, wherein the available signal length proportionally decreases (in linear fashion) with the scale of the coarse-graining procedure. Consequently, this imposes a constraint on the number of scales that can be reliably assessed. While ComEDA partially mitigates this limitation by exhibiting reliability even on ultra-short series, the user must still carefully select the maximum range of scale to be investigated. Moreover, the application of this framework to data from different emotionally charged contexts could facilitate the generalization of the results obtained. This would enable the portrayal of the affective state of a subject across a spectrum of conditions, thereby enriching our understanding of emotional responses in diverse scenarios. In this work, we applied for the first time the ComEDA algorithm in a multiscale fashion to assess the complexity of EDA response to emotion-eliciting stimuli. The results highlighted the importance of considering physiological phenomena at multiple time scales to better characterize emotional responses. Indeed, by increasing the range of the scale factor values at which we observed the spatial characteristics of the trajectories in the phase space, we were capable of discerning more nuances of emotional states, such as the difference between happiness and fear, both characterized by high arousal level, but opposite valence. An additional advantage of the MComEDA approach is the reliability in the application to ultra-short recordings, which allowed the multiscale analysis even starting from time series lasting less than one minute.

Future development of this research could be directed towards an in-depth study of the correlation between the levels of multiscale complexity of EDA signals and the scores of the arousal dimension acquired in different elicitation conditions. Furthermore, the evaluation of ultra-short EDA time series in new stimulation protocols able to objectively elicit more distinct stimuli in terms of arousal and valence could be a promising test, for example using virtual reality, which could induce a stronger emotional response [56]. The multiscale analysis could also bring interesting results in the clinical field, where long-term monitoring of the EDA signals could provide new information on stress management and its influence on circadian rhythm. This information is of great importance for practical applications such as telemedicine, enabling the naturalistic monitoring of patients’ autonomic status [57,58]. By utilizing recent advances in wearable sensor technology, we are moving towards a more accurate and non-invasive acquisition of these signals during daily life. This will aim to provide constant monitoring tailored to the individual and feedback for therapeutic approaches [59,60,61]. In addition, moving forward, we intend to adopt multivariate analysis, capitalizing on recent sensor innovations that support multiple signal acquisition modalities. This approach promises enhanced insights into autonomic function, thereby improving our ability to diagnose and manage medical conditions effectively [62,63,64].

## Figures and Tables

**Table 1:** Statistical analysis of *ComEDA* and *MComEDA* values obtained for the CASE dataset. Results of the statistical tests applied to MComEDA (**left**) and to ComEDA (**right**) values obtained from noiseless EDA signals of the CASE dataset. The values correspond to the *p*-values obtained from the Wilcoxon non-parametric test (α=0.05) after Bonferroni’s correction, comparing each stimulation outcome related to the emotion reported in the corresponding row against the column. The emotion labels are shortened with the following convention: “amu” = amusement, “bor” = boredom, “rel” = relaxation, and “sca” = scariness. Table cells in yellow indicate statistically significant results (*p*-value < 0.05). The *p*-value tables are symmetrical with respect to the diagonal, the gray cells correspond to self-matches or comparisons equal to those for which the *p*-value is already reported.

MComEDA					ComEDA					
	amu	bor	rel	sca			amu	bor	rel	sca
amu		0.001	7.310 × 10^−5^	0.024		amu		0.002	2.072 × 10^−4^	1.000
bor			1.000	4.797 × 10^−5^		bor			1.000	4.797 × 10^−5^
rel				5.990 × 10^−6^		rel				1.944 × 10^−5^
sca						sca				

**Table 2:** Statistical analysis of ComEDA and MComEDA values for the AMIGOS dataset. Results of the statistical tests applied to MComEDA (**left**) and to ComEDA (**right**) values obtained from noiseless EDA signals of the AMIGOS dataset. The values correspond to the *p*-values obtained from the Wilcoxon non-parametric test (α=0.05) after Bonferroni’s correction, comparing each stimulation outcome related to the valence–arousal quadrant reported in the corresponding row against the column. The quadrant labels are shortened with the following convention: “H” = High, “L” = Low, “A” = Arousal, “V” = Valence (e.g., HALV refers to the quadrant of high arousal and low valence). Table cells in yellow indicate statistically significant results (*p*-value < 0.05). The *p*-value tables are symmetrical with respect to the diagonal, the gray cells correspond to self-matches or comparisons equal to those for which the *p*-value is already reported.

MComEDA					ComEDA					
	HAHV	LAHV	LALV	HALV			HAHV	LAHV	LALV	HALV
HAHV		1.000	0.007	0.857		HAHV		0.2712	0.154	0.879
LAHV			0.606	0.024		LAHV			1.000	0.004
LALV				3.930 × 10^−4^		LALV				0.004
HALV						HALV				

**Table 3:** Statistical analysis of EDASymp values for the CASE (**left**) and AMIGOS (**right**) datasets. Results of the statistical tests applied to the EDASymp values obtained from noiseless EDA signals of both datasets. The values correspond to the *p*-values obtained from the Wilcoxon non-parametric test (α=0.05) after Bonferroni’s correction, comparing each stimulation outcome related to the valence–arousal quadrant reported in the corresponding row against the column. The emotion labels are shortened with the following convention: “amu” = amusement, “bor” = boredom, “rel” = relaxation, and “sca” = scariness. The quadrant labels for the AMIGOS dataset are shortened with the following convention: “H” = High, “L” = Low, “A” = Arousal, “V” = Valence (e.g., HALV refers to the quadrant of high arousal and low valence). Table cells in yellow indicate statistically significant results (*p*-value < 0.05).The *p*-value tables are symmetrical with respect to the diagonal, the gray cells correspond to self-matches or comparisons equal to those for which the *p*-value is already reported.

CASE Dataset					AMIGOS Dataset					
	amu	bor	rel	sca			HAHV	LAHV	LALV	HALV
amu		1.000	1.000	1.000		HAHV		0.144	0.129	0.254
bor			1.000	0.124		LAHV			0.003	1.000
rel				0.004		LALV				1.647 × 10^−4^
sca						HALV				

## Data Availability

The data of the CASE dataset used in this study are available in the repository at https://gitlab.com/karan-shr/case_dataset [38] (accessed on 10 September 2023). The data of the AMIGOS dataset are available at https://www.eecs.qmul.ac.uk/mmv/datasets/amigos/index.html (accessed on 1 March 2024), upon the authors’ consent [39]. The Matlab code for ComEDA index estimation is publicly available online at https://github.com/NardelliM/ComEDA (accessed on 10 October 2023).

## Abbreviations

The following abbreviations are used in this manuscript:

EDA	Electrodermal Activity
HRV	Heart Rate Variability
ANS	Autonomic Nervous System
MComEDA	Multiscale ComEDA
CASE	Continuously Annotated Signals of Emotion
AMIGOS	Affect, Personality and Mood Research on Individuals and Groups
HVHA	High Valence–High Arousal
HVLA	High Valence–Low Arousal
LVHA	Low Valence–High Arousal
LVLA	Low Valence–Low Arousal
